# Examining the quality of life among pregnant women diagnosed with gestational diabetes mellitus: A systematic review and meta-analysis for women’s health promotion

**DOI:** 10.34172/hpp.2024.05

**Published:** 2024-07-29

**Authors:** Majid Mobasseri, Mehrnoush Mobasseri, Ayda Alizadeh, Sara Hakimzadeh, Seyedeh Sara Ebadi, Samin Imani, Nima Pourgholam, Saber Azami-Aghdash

**Affiliations:** ^1^Endocrine Research Center, Tabriz University of Medical Sciences, Tabriz, Iran; ^2^Student Research Committee, Tabriz University of Medical Sciences, Tabriz, Iran; ^3^School of Nursing and Midwifery, Iran University of Medical Science, Tehran, Iran; ^4^Research Center for Evidence-based Medicine, Iranian EBM Centre: A JBI Centre of Excellence, Faculty of Medicine, Tabriz University of Medical Sciences, Tabriz, Iran

**Keywords:** Gestational diabetes mellitus, Quality of life, Pregnant women, Women’s health, Systematic review and meta-analysis

## Abstract

**Background:**

Quality of life (QoL) of women with gestational diabetes mellitus (GDM) is one of the fundamental issues and public health challenges. This study examines the QoL among pregnant women with GDM through a systematic review and meta-analysis.

**Methods:**

A search was conducted in Scopus, PubMed, and the Web of Science databases for articles published until Jan 30, 2024. Manual searches of gray literature, Google Scholar, reference checks, and citation checks were conducted. The JBI’s Critical Appraisal Checklist for Analytical Cross-Sectional Studies was utilized to assess the quality of the articles’ reporting. The random model implemented in Stata software (version 16; Stata Corp.) was utilized to conduct the meta-analysis.

**Results:**

Among the 516 studies obtained from the literature, only 15 were deemed suitable for inclusion. Most studies (73.3%) were conducted in nations with high-income levels. Additionally, general QoL was assessed in most studies (11 studies). The SF-36 and WHOQOLBREF questionnaires were the most often utilized. Based on the SF-36 measure, there was no statistically significant difference in the QoL of patients with GDM compared to the control group in most of dimensions. The WHOQOL-BREF instrument was utilized to estimate the QoL score at 49.69. The EQ-5D-5L tool revealed a difference in QoL scores between the GDM and control groups (MD=-7.40). The research findings were highly heterogeneous. The median evaluation score for the reporting quality of the articles was calculated to be 5, with a mean of 4.8 out of 7.

**Conclusion:**

The results of the present study showed that GDM reduces the QoL of pregnant women, especially in terms of mental and social health. Therefore, interventions and support programs should be designed and implemented to improve these women’s QoL.

## Introduction

 Chronic diseases are a significant concern in modern society, both in terms of health and treatment. Diabetes is a prevalent chronic condition and a significant health concern worldwide. ^1-3^ This metabolic ailment commonly presents as asymptomatic during its initial phases, with its primary manifestation being chronic hyperglycemia. This condition leads to the development of diseases and damage in multiple organs concurrently with the elevation of blood sugar levels.^4^ Gestational diabetes mellitus (GDM) is a significant kind of diabetes that raises the likelihood of diabetes in the mother after pregnancy, as well as causing difficulties for both the mother and the fetus during and after pregnancy.^5-7^ The occurrence of GDM ranges between 1%-14% during pregnancy, and this variation is influenced by factors such as geographical location, characteristics of the population being investigated, variations in data collection methods, non-random selection of women, and the diagnostic criteria employed.^8,9^

 The quality of life (QoL) of people with GDM is an essential and fundamental concern.^10^ QoL is a multidimensional, subjective, complex concept and a complete and adaptable process encompassing all areas of people’s lives. In other words, QoL is a distinct individual perception and a means of expressing one’s thoughts regarding health or other aspects of life. This procedure is evaluated using standardized instruments to analyze people’s viewpoints.^11-13^ It is critical to take into account the broad and detrimental impacts of GDM on pregnant women’s QoL since the disease’s progression and lack of therapy will harm patients’ QoL in numerous ways.^14,15^

 In recent years, numerous studies on the impact of GDM on the QoL of expectant women have been conducted; however, these studies were limited in scope and utilized a smaller sample size. Moreover, based on the results of the results of initial literature review and our best knowledge, despite the limited number of literature reviews available in this field, so far, a systematic review and meta-analysis study that specifically and comprehensively evaluated the issue of QoL among pregnant women with GDM is not available. As a result, they cannot offer precise and helpful information for macro decision- and policy-making. A methodical summary of the findings of these investigations can obtain the data required for macro-level policy and decision-making. The current study is a meta-analysis and systematic review of QoL in pregnant women with GDM.

## Materials and Methods

 This study is a systematic review and meta-analysis designed and conducted to estimate the QoL of pregnant women with GDM and the impact of GDM on the QoL of mothers in 2023. Preferred Reporting Items for Systematic Reviews and Meta-Analyses (PRISMA) was used as the guideline for this study. ^16^

###  Search strategy

 The current study’s search strategy was developed and implemented by an experienced and highly knowledgeable librarian under the supervision of a subject matter expert (Supplementary file 1 - Search strategy). The necessary information was gathered by searching related terms in Mesh in PubMed, Scopus, and the Web of Science databases. Articles published up until January 30, 2024, were searched. In addition, we ran a manual search in Google Scholar to discover and include more published works. Following the removal of publications with poor relationships to the study’s aims and the selection of original papers, additional steps were taken to ensure the validity of identifying and reviewing the existing articles. These steps included checking references, citations, and gray literature.

###  Inclusion and exclusion criteria

####  Inclusion criteria

- The analysis included all international publications published in English that discussed the QoL of mothers with GDM and the effect of GDM on the QoL of pregnant women.

####  Exclusion criteria

- Studies that did not report quantitative measures of QoL status.
- Interventional studies aimed at improving the QoL of pregnant women with GDM
- Research and reports that do not provide full text or that are inaccessible
- Studies presented at conferences

###  Selection/screening of studies

 Two research team members conducted the entire article selection and screening process autonomously. The initial phase involved resolving disputed cases through discussion. If further skill and information were needed, the cases were forwarded to a third party. First, all article titles were examined, and those that did not align with the study’s goals were removed. Next, studies deemed inappropriate or irrelevant were eliminated after thoroughly analyzing the papers’ abstracts and full texts. Endnote X5 was utilized for organization, duplicate detection, and title and abstract verification. Utilizing a PRISMA (Preferred Reporting Items for Systematic Reviews and Meta-Analyses) flowchart, the selection and screening procedure outcomes were documented.

###  Evaluation of the reporting quality of articles

 Using the JBI’s Critical Appraisal Checklist for Analytical Cross-Sectional Studies, two evaluators independently evaluated each article’s reporting quality during the full-text screening stage. JBI’s critical evaluation tools aim to assess a study’s methodological quality and ascertain how well it has handled the potential for bias in its planning, execution, and analysis. The cross-sectional analysis study evaluation tool consists of eight questions. The third question of the instrument, “Was the exposure measured validly and reliably?” was eliminated because it did not apply to any of the case studies in the current study. Instead, seven questions were used to evaluate the articles. The options for this instrument are yes, no, opaque, and unrelated. The consensus of two evaluators determined each article’s final evaluation score, which ranged from 0 to 7 (the number of “yes” options). In order to resolve disagreements between the two evaluators, a third evaluator was consulted.

###  Extracting the data

 Using Microsoft Office Word 2013, a manual data extraction form was first created to extract the data. Author, year, first author affiliation (country), study goal, participants, sample size, age mean, QoL type (HRQoL or GQoL), instrument, and QoL score are among the extracted data.

 First, the data from 3 articles were experimentally extracted for these forms, and the deficiencies and problems in the initial form were resolved. Two people independently extracted the information, and ambiguous cases were resolved by consulting the research team members.

###  Data analysis methods

 Using a random model and meta-analysis statistical techniques, the QoL of pregnant women with GDM was estimated. The meta-analysis used Stata software (Stata Corp., version 16). ^17^ The results were shown using forest plot charts, in which the size of each square represents the sample size, and the confidence range of around 95% for each research is represented by the lines drawn on either side of the square. The study’s results’ heterogeneity was quantified using the I^2^ index. I^2^ values below 50% were regarded as low heterogeneity, those between 50% and 74% as medium heterogeneity, and those above 75% as high heterogeneity.^18^

 Additionally, subgroup analyses based on QoL dimensions were carried out. The analysis for publication bias potential was conducted using funnel plot diagrams and Egger’s regression test, with a significance level of 0.1%.^19^ The trim-and-fill test was not applied since there was little chance of publication bias.

 The remaining data was summarized using descriptive statistics, specifically the mean, percentage, and frequency, employing Microsoft Office Excel 2010. Additionally, graphics were created using Microsoft Office Excel 2010.

## Results

###  The results of screening articles

**Figure 1:**
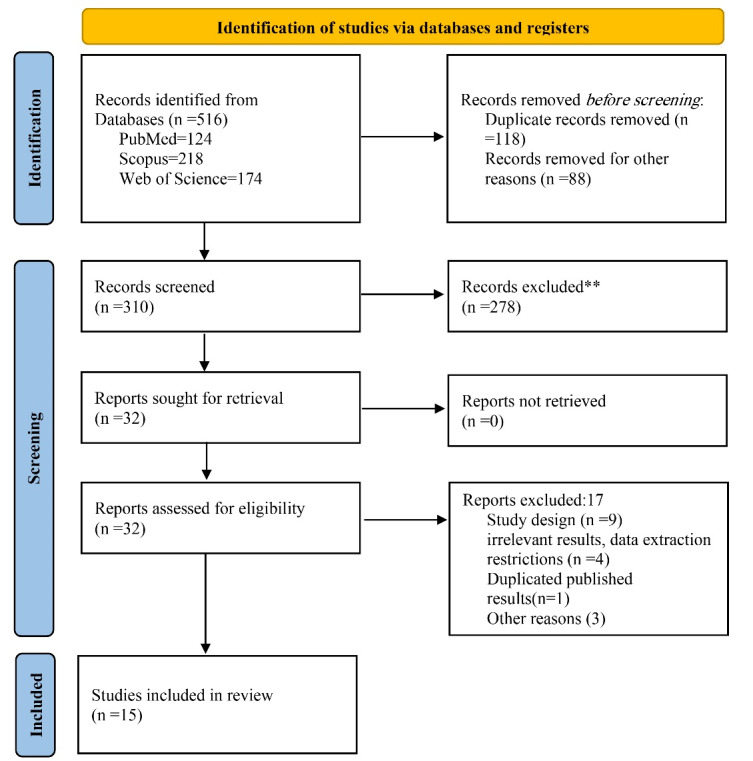
Screening process of studies on the QoL of pregnant women with GDM

 Finally, 516 studies were found through database searches. After using Endnote X5 software to eliminate duplicate articles and for other purposes, 310 articles made it to the screening phase. During the initial phase, 278 articles were eliminated as they were deemed irrelevant by two researchers who independently evaluated the titles and abstracts of the articles. In the second phase, the researchers read the complete text of the remaining articles. They rejected 17 others because they were irrelevant or did not fit the inclusion criteria, leaving 15 papers for analysis (Figure 1).

###  Study specifications

 The studies under examination were carried out in 12 nations, with Poland having the highest number of studies, specifically 3. The articles were published between 2002 and 2023, with a median publication year of 2018. The analysis contained a total sample size of 15 publications, comprising 16 155 individuals (3216 in the GDM group and 12 939 in the control group). The participants had an average age of 31.6 years. According to the World Bank’s income-based classification of countries, most studies (73.3%) were done in countries classified as high-income. Four studies assessed HRQoL, and 11 research studies assessed GQoL. Across the 15 reviewed papers, a total of 18 instruments were utilized. The most commonly employed instruments were SF-36 and WHOQOL-BREF (Table 1).

**Table 1:** Data extraction table related to examining the quality of life among pregnant women diagnosed with gestational diabetes mellitus

**Author, year**	**Country**	**Aim of study**	**Participants (N)**	**Mean age**	**Type of QoL (HRQoL or GQoL)**	**Tool**	**QOL score**
Sayed Shama et al,^20^ 2020	Egypt	Explore QOL among women with GDM	GDM (200)	29.7 ± 5.8	GQoL	QoL index diabetes version-111	Social/economic: 66%, Psychological/spiritual: 64%, Family: 48%, Health and functioning: 32%, Total: 43%
Halkoaho et al,^21^ 2010	Finland	Investigate the effects of GDM on women’s HRQoL after delivery	GDM (77) Control (54)	-	HRQoL	15D HRQoL	GDM: 0.938, Control: 0.931
Lee et al,^22^ 2020	Malaysia	Identify factors associated with poor-to-moderate QOL among women with GDM	GDM (526)	32	GQoL	AsianDQoL	Median: 87.62 (IQR 9.52) (21-105)
Pantzartzis et al,^23^ 2019	Greece	investigate the effect of GDM on the QoL of pregnant women during the third trimester of pregnancy	GDM (31) Control (31)	32.9 ± 5.2	HRQoL	EQ-5D-5L	GDM: 75.5 ± 19.3, Control: 88.1 ± 8.0
Pantzartzis et al,^23^ 2019	Greece	investigate the effect of GDM on the QoL of pregnant women during the third trimester of pregnancy	GDM (31) Control (31)	32.9 ± 5.2	HRQoL	WHOQOL-BREF	*Physical QoL:* GDM: 25.5 ± 5.1, Control: 27.5 ± 4.0 *Psychological QoL:* GDM: 22.3 ± 3.2, Control: 22.9 ± 2.4 *Social relationships:* GDM: 12.0 ± 2.7, Control: 12.8 ± 2.6 *Social environment:* GDM: 28.5 ± 3.9, Control: 30.8 ± 3.4
Pantzartzis et al,^23^ 2019	Greece	investigate the effect of GDM on the QoL of pregnant women during the third trimester of pregnancy	GDM (31) Control (31)	32.9 ± 5.2	HRQoL	ADDQoL	Excellent (3.2%), Very good (35.5%), Good (32.3%), Neither good nor bad (16.1%), Bad (6.5%), Very bad (6.5%)
Danyliv et al,^24^ 2015	Ireland	Examined HRQOL in a group of women who had GDM in the index pregnancy 2 to 5 years previously and compared it to a group of women with NGT	GDM (111) NGT (231)	38.15	HRQoL	EQ- 5D-3 L (with VAS component)	GDM: 80.31 (SE 1.36), NGT: 83.98 (SE 0.79)
Liu et al,^25^ 2020	China	examine impacts of GDM on QoL domains in Chinese pregnant women	GDM (969) Control (12389)	29.5 ± 3.2	>GQoL	SF-36	*Physical functioning:* Control:75 (60–85), GDM: 75 (60–85) *Role physical:* Control: 50 (25–100), GDM:50 (25–100) *Bodily pain:* Control: 84 (74–100), GDM: 84 (74–100) *General health:* Control: 87 (72–97), GDM: 85 (72–95) *Vitality:* Control: 80 (70–85), GDM: 80 (70–85) *Social functioning:* Control: 89 (78–100), GDM: 89 (78–100) *Role emotional:* Control: 100 (67–100), GDM: 100 (50–100) *Mental health:* Control: 80 (72–88), GDM: 80 (68–88) *Physical component summary:* Control: 45 (40–51), GDM: 45 (39–51) *Mental component summary:* Control: 57 (50–62), GDM: 56 (50–62)
Kutowska et al,^26^ 2012	Poland	Evaluate the life quality level among women who had GDM	GDM (100)	30.9	GQoL	subjective assessment (10-point: 1 poor, 10–very good)	6.9
Doğan and Beji,^27^ 2023	Turkey	determine the QoL and depression of women with GDM during pregnancy and the postpartum period	GDM (100) Control (100)	30 ± 6.79	GQoL	SF-36	*Physical functioning:* Control: 52.45 ± 14.48, GDM: 54.95 ± 12.88 *Role physical:* Control: 16.25 ± 18.59, GDM: 18.75 ± 26.44 *Bodily pain:* Control: 35.6 ± 18.54, GDM: 35.5 ± 17.19 *General health:* Control: 42.90 ± 11.72, GDM: 42.25 ± 10.94 *Vitality:* Control: 38.05 ± 11.05, GDM: 38.95 ± 13.39 *Social functioning:* Control: 28 ± 29.85, GDM: 40 ± 14.93 *Role emotional:* Control: 28 ± 29.85, GDM: 20.66 ± 27.53 *Mental health:* Control: 51.24 ± 8.78, GDM: 49.92 ± 11.37
Kopec et al,^28^ 2015	Poland	Describe changes in patient-reported outcomes in women with GDM	GDM (205)	30.9 ± 4.5	GQoL	SF-36	*Baseline:* Physical component: 49.7, Mental component: 48.5 *Follow-up:* Physical component: 47.5, Mental component: 48.8
Dalfrà et al,^29^ 2012	Italy	Evaluation of QoL in pregnant women with GDM	GDM (176) Control (39)	33.9 ± 4.5	GQoL	SF-36	*Physical functioning:* Control: 65.3 (24.6), GDM: 68.0 (25.5) *Role physical:* Control: 20.0 (29.8), GDM: 43.4 (41.2) *Bodily pain:* Control: 60.5 (22.8), GDM: 77.3 (23.7) *General health:* Control: 79.8 (16.5), GDM: 74.3 (15.3) *Vitality:* Control: 57.4 (16.9), GDM: 56.8 (17.3) *Social functioning:* Control: 70.5 (22.1), GDM: 72.5 (21.8) *Role emotional:* Control: 71.1 (34.8), GDM: 66.1 (39.5) *Mental health:* Control: 74.4 (18.2),GDM: 71.2 (17.3) *Physical component:* Control: 40.1 (8.0), GDM: 44.8 (9.5) *Mental component:* Control: 51.1 (10.2), GDM: 48.1 (10.5)
Mautner et al,^30^ 2009	Austria	Explore the influence of hypertensive disorders, GDM, and preterm birth as risk factors for HRQL and depressive symptoms during late pregnancy and postpartum	GDM (11)	30.4 (5.73)	HRQoL	WHOQOL-BREF	Physical QoL: 78.19 (10.81), Psychological QoL: 73.11 (19.40), Social relationships: 81.06 (22.39), Social environment: 76.54 (14.29), Global: 71.59 (15.90)
Iwanowicz-Palus et al,^31^ 2019	Poland	Evaluate levels of QoL, social support, acceptance of illness, and self-efficacy among pregnant patients with hyperglycemia	GDM (339)	-	GQoL	WHOQOL-BREF	Physical QoL: 12.60 (1.71), Psychological QoL: 14.92 (2.36), Social relationships: 15.21 (2.52), Social environment: 14.88 (2.35), General QoL: 3.64 (0.88), General Health: 3.43 (0.83)
Baneh et al,^32^ 2018	Iran	Determine the relationship between the acceptance of illness and QoL in mothers with GDM	GDM (150)	31.21 ± 5.97	GQoL	SF-36	Physical component: 47.6 ± 6.78, Mental component: 40.64 ± 4.88, Total QoL: 87.7 ± 8.3
Mokhlesi et al,^33^ 2019	Iran	Evaluate the QOL of mothers with GDM and its associated factors using a specific questionnaire	GDM (200)	31.85 (5.34)	GQoL	GDMQ-36	*Mean score based on 100:* Concerns about a high-risk pregnancy: 31.46 (19.82) Medication and treatment: 42.79 (15.47) Perceived constraints: 41.85 (17.61) GDM complications: 50.77 (24.80) Support: 76.52 (14.57) Total: 46.83 (11.66)
Rumbold and Crowther,^34^ 2002	Australia	Assess if women screening positive for GDM will experience a reduction in their Qol	GDM (21) Control (95)	30 (4)	GQoL	SF-36	*Physical functioning:* Control: 48 (21), GDM: 52 (25) *Role physical:* Control: 30 (39), GDM: 26 (38) *Bodily pain:* Control: 59 (22), GDM: 63 (23) *General health:* Control: 75 (21), GDM: 71 (13) *Vitality:* Control: 47 (19), GDM: 56 (17) *Social functioning:* Control: 75 (21), GDM: 69 (21) *Role emotional:* Control: 76 (37), GDM: 79 (37) *Mental health:* Control: 77 (16), GDM: 80 (10)

Abbreviations: GDM, gestational diabetes mellitus; GQoL, general quality of life; HRQoL, health-related QoL; ADDQoL, Audit of Diabetes Dependent Quality of Life; NGT, normal glucose tolerance; SF-36, 36-Item Short-Form Health Survey.

###  Results of a meta-analysis of QoL based on the SF-36 tool

**Figure 2:**
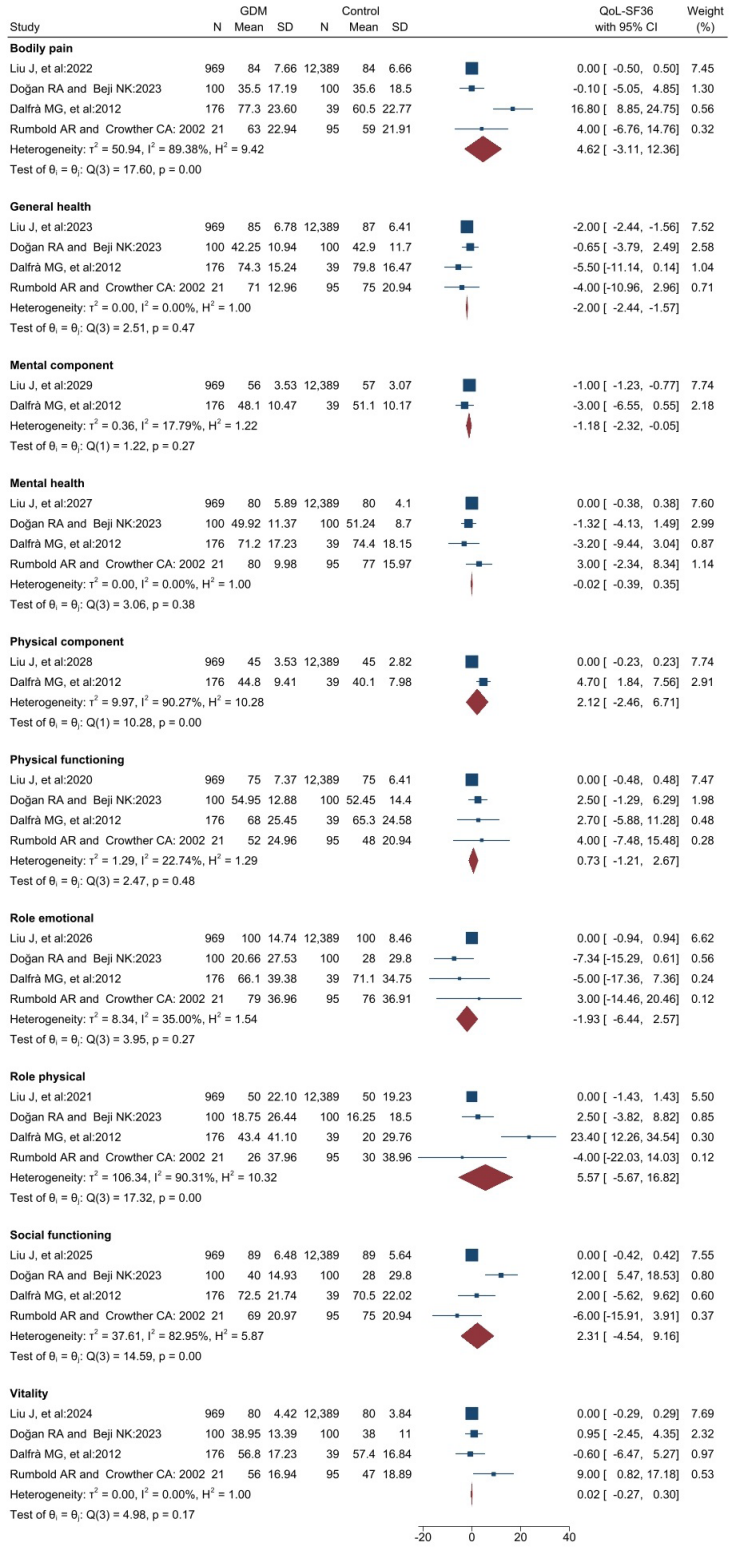
The meta-analysis results comparing the QoL of GDM patients with control groups using the SF-36 tool

**Figure 3:**
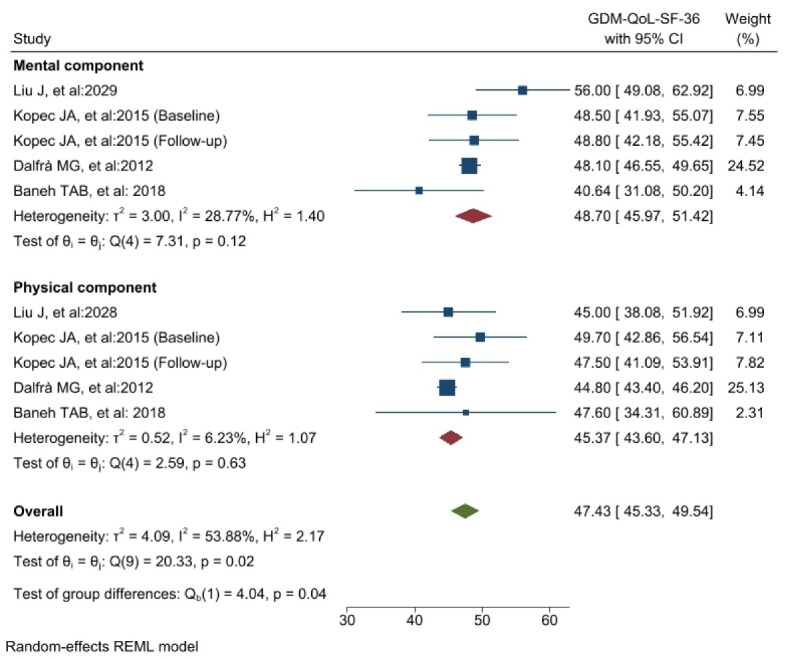
The results of the meta-analysis of the QoL of patients with GDM regarding Physical and Mental components with the SF-36 tool

 Comparing the QoL of patients with GDM with the control groups showed that in two subgroups, “general health” and “mental component,” the QoL was significantly lower in the group of patients with GDM. In other dimensions, no significant differences were observed between the two groups (Figure 2). The results showed that there is high heterogeneity in the four dimensions (I^2^ = above 75%), Baneh et al^32^ and Kopec et al^28^ only documented the outcomes of the “physical component” and “mental component” dimensions within a cohort of GDM patients. As a result, the findings of these two investigations and other research that reported on these two dimensions were evaluated independently in the GDM group. The findings indicate that the mean QoL for the “mental component” is 48.7 [45.9-51.4 with 95% CI], while for the “physical component” it is 45.3 [43.6-47.1 with 95% CI] **(**Figure 3**).**

**Figure 4:**
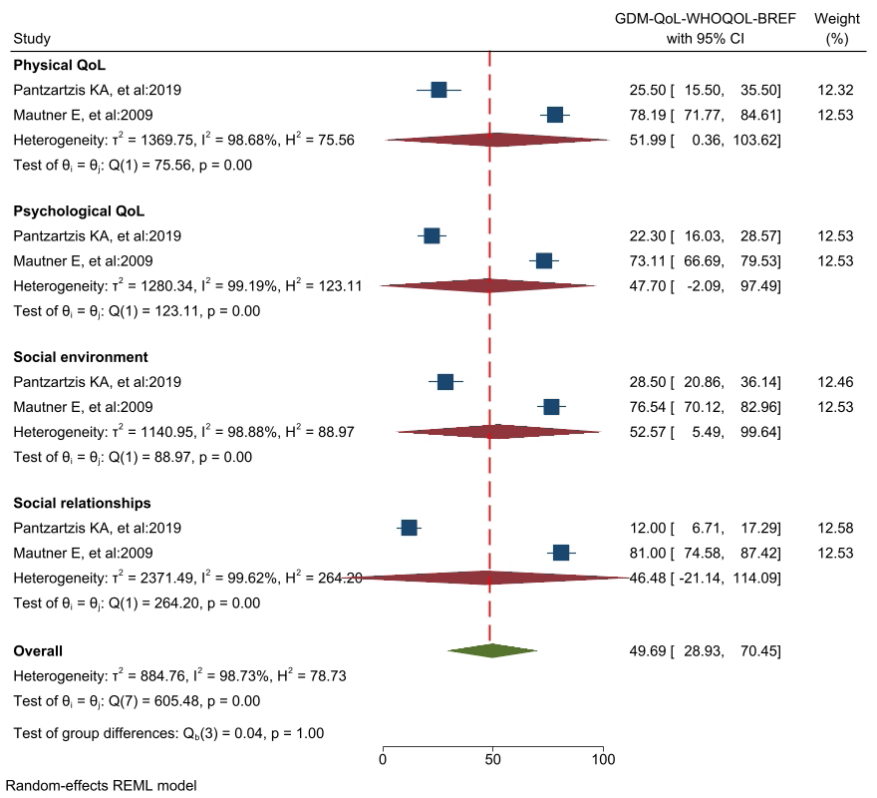
The results of the meta-analysis of the QoL of GDM patients using the WHOQOL-BREF tool

**Figure 5:**
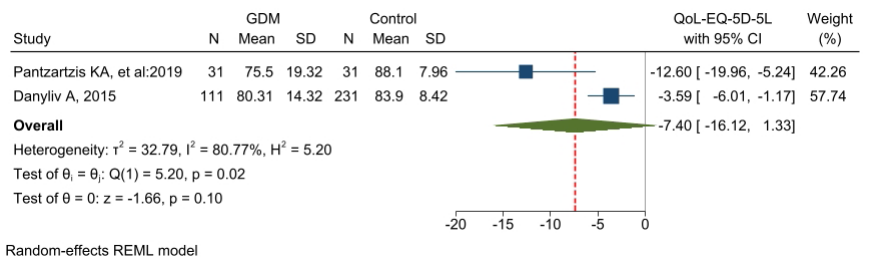
The meta-analysis results comparing the QoL of GDM patients with control groups using the EQ-5D-5L tool

###  The results of the meta-analysis of QoL based on the WHOQOL-BREF tool

 Meta-analysis-appropriate information was found exclusively in the results of two articles analyzed using this tool. The meta-analysis revealed that the QoL score is 49.69 [95% CI: 28.93-70.45]. The “social environment” dimension received the highest score (52.57), while the “social relationships” dimension received the lowest score (46.48) (Figure 4). Additionally, the study’s findings exhibited considerable heterogeneity (I^2^ = 98.7%). The study’s results did not indicate publication bias (Egger’s test, *P* = 0.584, Z = -0.55) (see Supplementary file 2, Funnel Plot Diagram A). The instrument in question was also utilized in the research conducted by Iwanowicz-Palus et al^31^ in Hungary, which involved 339 patients diagnosed with GDM. Nevertheless, their research was excluded from our meta-analysis due to a scoring methodology deviation from the other studies. It was discovered that the participants’ subjective assessment of their overall health score of 3.43 was lower than their actual QoL score of 3.64.

###  The results of the meta-analysis of QoL based on EQ-5D-5L tool

 The results of two studies conducted using this instrument, each with a sample size of 262 individuals in the control group and 142 individuals in the GDM group, were incorporated into the meta-analysis. Figure 5 shows that the difference between the two groups was not statistically significant (MD = -7.40 [-16.12-1.33]). The heterogeneity test indicated that the study’s findings exhibited significant heterogeneity (I^2^ = 80.7%). Furthermore, there was no likelihood of publication bias in the findings (Egger’s test, *P* = 1 Z = -0.00) (Supplementary file 2, Diagram B - Funnel Plot).

###  The results based on other tools

 Sayed Shama et al conducted a study in Egypt involving 200 patients and employing the Quality of Lile index diabetes version-111 instrument. The findings revealed that approximately 43% of the patients reported a satisfactory QoL, whereas 55% reported a low QoL.^20^ The findings of a study conducted in Finland by Halkoaho et al utilized the 15D HRQoL tool to compare the QoL of 77 GDM patients with that of 55 healthy individuals. The QoL of the GDM group was 0.938, while the QoL of the control group was 0.931. These results indicated no statistically significant difference in QoL between the two groups.^21^ Using the AsianDQoL instrument, Lee et al assessed the QoL of 526 GDM patients in Malaysia. The mean QoL score of the participants was 87.62 (IQR 9.52) (105–21).^22^ Kutowska et al conducted a study using a questionnaire to objectively assess the QoL of 100 patients on a scale of 1 to 10. The patients rated their QoL as 6.9 out of 10.^26^ Mokhlesi et al conducted a study in Iran to assess the QoL of 200 expectant women diagnosed with GDM using the GDMQ-36 instrument. The results indicated that the patients’ overall QoL was 46.8 out of 100. Fear of high-risk pregnancy received the lowest score of 31.4.^33^

###  Scores for evaluating articles’ quality of reporting 

 The articles’ reporting quality was assessed with a median score of 5 and an average of 4.8 out of 7. The primary shortcomings of the articles pertained to their failure to account for confounding variables and implement appropriate measures to mitigate their influence. In addition, most of the articles failed to report their findings using appropriate statistics. A significant proportion of the articles relied on descriptive statistics. In contrast, applying advanced and effective analytical statistics, such as regression, was noticeably limited (see Supplementary file 3 for the results of the article’s reporting quality assessment).

## Discussion

 Finally, 516 research were extracted by searching databases, and 15 publications were included in the study. The majority of the studies were carried out in high-income nations. The general QoL was assessed in most investigations (11 studies). The most frequently used instruments were the SF-36 and WHOQOL-BREF. Using the SF-36 to compare the QoL of GDM patients to that of the control groups, the results indicated that the difference between the two groups was not statistically significant in most of dimensions. Using the WHOQOL-BREF measure, the QoL score was 49.69 (28.93-70.45 with 95% CI). The EQ-5D-5L tool revealed a difference in QoL scores between the GDM and control groups (MD = -7.40 [-16.12-1.33]).

 As previously stated, most of the studies were conducted in high-income nations. Most likely, studies undertaken in middle- and low-income nations could not be published in prestigious overseas journals or were removed from databases. Given that high-income countries appear to have a higher aging rate^35^ and that birth and youth populations are significant concerns, it stands to reason that these nations devote more resources to researching and addressing the health of pregnant women. Middle and low-income countries have a rate of old-age growth that is the same as high-income countries.^36^ In addition, these nations have significantly emphasized adolescent population growth in recent years. As a result, pregnancy health concerns and pregnancy-related complications, including GDM, must be given significant attention and priority. It is recommended that high-income nations, the World Health Organization, non-governmental organizations (NGOs), and related groups assist low- and middle-income nations in improving the QoL of pregnant women with GDM and resolving their associated issues since these countries’ governments and guardian organizations are unable to offer comprehensive support to pregnant women.

 The majority of the studies (11 studies) assessed GQoL. Although GQoL and HRQoL are frequently used interchangeably, they are distinct and separate concepts. GA person’s perceived QoL, or the evaluation of welfare or lack thereof, is called their GQoL. This is a multifaceted and typically more extensive term. While GQoL encompasses all emotional, social, and physical aspects of an individual’s existence, HRQoL is defined as evaluating how a disease, disability, or disorder impacts an individual’s health over time.^37-40^ Hence, upcoming research should emphasize HRQoL measurement, given that GDM is a specialized health and medical concern, and thus, such data is more pertinent and specific.

 Using the SF-36 to compare the QoL of patients with GDM to that of control groups revealed no statistically significant difference between the two groups, and the QoL of patients with GDM was also slightly higher. One plausible explanation for this phenomenon may be attributed to the instrument’s characteristics in question, which is to assess the GQoL of patients. Due to the numerous confounding variables in the lives of patients with GDM, it is challenging to determine whether GDM or other factors significantly impact QoL. The articles’ primary deficiency was the omission of instructions on identifying and controlling confounding variables, which was also reflected in evaluating the quality of the reporting. When examining the difference in QoL scores between the control and GDM groups as measured by the EQ-5D-5L tool, we found that the QoL is substantially lower in the GDM group. This matter may also indicate bias in the research findings employing publicly available instruments of GQoL. In an article discussing the difficulties of assessing QoL, Lin et al identified this as one of the fundamental obstacles.^41^ Consequently, precision in determining and quantifying the form of QoL can significantly influence the outcomes of assessments.

 After examining the various dimensions measured by various research instruments, it is evident that the QoL of GDM patients is significantly compromised in the areas of mental health and social functioning, while the physical dimensions remain relatively unaffected. This might be because most of the research was conducted in high-income nations, where healthcare is generally of higher quality. Consequently, it is more critical and pressing to focus on developing treatments that seek to enhance people’s QoL in social and psychological aspects.^42,43^

 Considering the diversity and variations in the instruments employed for assessing the QoL is crucial. An essential component of this topic revolves around categorizing tools as specific or general. Only a limited number of articles in this analysis employed precise measuring instruments. The study conducted by Mokhlesi et al in Iran stands out as the sole research that employed a specialized tool, GDMQ-36, to assess QoL in 200 pregnant women diagnosed with GDM.^33^ The tool comprises 36 questions that assess the QoL of pregnant women with GDM across five dimensions.^33^ Specialized instruments focus on studying a particular population or condition and have numerous applications in clinical and research settings. Hence, it is strongly advised to incorporate general and specialized tools to achieve comprehensive research outcomes. Furthermore, it is crucial to consider not only general aspects but also the specific characteristics of the disease and the unique conditions of each patient group.^44^

 Unlike the few review studies that have been conducted in this area, this study aimed to combine (meta-analyze) quantitatively the study results in order to present comprehensive and transparent information to decision-makers, healthcare providers, expectant mothers, researchers, and readers based on the findings of the literature search and the researchers’ personal experiences.^45,46^ Nevertheless, it is essential to acknowledge that the current study has significant limitations. Esteemed readers are advised to approach the study’s findings cautiously when examining, interpreting, and applying them to conclude. A primary limitation of this study was its exclusive analysis of studies conducted in the English language. Including research published in other languages in the analysis could have yielded different results. One additional constraint of the present analysis was the small number of studies included in each subgroup, which could potentially bias the study’s conclusions. Also, the results of the heterogeneity test showed a high heterogeneity in the results of the studies. The two main reasons for this could be the small number of studies analyzed in each of the subgroups and the differences between countries due to the countries’ different economic and social structures.

## Conclusion

 The results of the present study showed that GDM significantly reduces the QoL of pregnant women, especially in terms of mental and social health. Therefore, interventions and support programs should be designed and implemented to improve these people’s QoL. However, highly accurate measures utilizing specialized and valid tools are required, particularly for health-related QoL. It is imperative to design and implement epidemiological and interventional studies in low and middle-income countries to understand the current situation and try to improve it.

## Competing Interests

 The authors declare that they have no competing interests.

## Ethical Approval

 The study was approved by the medical ethics committee of Tabriz University of Medical Sciences, Iran (Registration No: IR.TBZMED.REC.1402.565).

## Supplementary Files


Supplementary file 1. Search Strategy

Supplementary file 2. Funnel charts to measure the possibility of publication bias in the results of articles

Supplementary file 3. The results of the evaluation of the quality of reporting articles

## References

[1] Zimmet PZ, Magliano DJ, Herman WH, Shaw JE (2014). Diabetes: a 21st century challenge. Lancet Diabetes Endocrinol.

[2] Mohseni M, Shams Ghoreishi T, Houshmandi S, Moosavi A, Azami-Aghdash S, Asgarlou Z (2020). Challenges of managing diabetes in Iran: meta-synthesis of qualitative studies. BMC Health Serv Res.

[3] Mobasseri M, Shirmohammadi M, Amiri T, Vahed N, Hosseini Fard H, Ghojazadeh M (2020). Prevalence and incidence of type 1 diabetes in the world: a systematic review and meta-analysis. Health Promot Perspect.

[4] Roglic G (2016). WHO global report on diabetes: a summary. Int J Noncommun Dis.

[5] McIntyre HD, Catalano P, Zhang C, Desoye G, Mathiesen ER, Damm P (2019). Gestational diabetes mellitus. Nat Rev Dis Primers.

[6] Buchanan TA, Xiang AH, Page KA (2012). Gestational diabetes mellitus: risks and management during and after pregnancy. Nat Rev Endocrinol.

[7] Di Bernardo SC, Lava SA, Epure AM, Younes SE, Chiolero A, Sekarski N (2023). Consequences of gestational diabetes mellitus on neonatal cardiovascular health: MySweetHeart Cohort study. Pediatr Res.

[8] Jafari-Shobeiri M, Ghojazadeh M, Azami-Aghdash S, Naghavi-Behzad M, Piri R, Pourali-Akbar Y (2015). Prevalence and risk factors of gestational diabetes in Iran: a systematic review and meta-analysis. Iran J Public Health.

[9] Paulo MS, Abdo NM, Bettencourt-Silva R, Al-Rifai RH (2021). Gestational diabetes mellitus in Europe: a systematic review and meta-analysis of prevalence studies. Front Endocrinol (Lausanne).

[10] Ansarzadeh S, Salehi L, Mahmoodi Z, Mohammadbeigi A (2020). Factors affecting the quality of life in women with gestational diabetes mellitus: a path analysis model. Health Qual Life Outcomes.

[11] Costa DS, Mercieca-Bebber R, Rutherford C, Tait MA, King MT (2021). How is quality of life defined and assessed in published research?. Qual Life Res.

[12] Azami-Aghdash S, Pournaghi-Azar F, Moosavi A, Mohseni M, Derakhshani N, Alaei Kalajahi R (2021). Oral health and related quality of life in older people: a systematic review and meta-analysis. Iran J Public Health.

[13] Maleki MR, Derakhshani N, Azami-Aghdash S, Naderi M, Nikoomanesh M (2020). Quality of life of people with HIV/AIDS in Iran: a systematic review and meta-analysis. Iran J Public Health.

[14] Minschart C, De Weerdt K, Elegeert A, Van Crombrugge P, Moyson C, Verhaeghe J (2021). Antenatal depression and risk of gestational diabetes, adverse pregnancy outcomes, and postpartum quality of life. J Clin Endocrinol Metab.

[15] Liang CC, Chao M, Chang SD, Chiu SY (2020). Impact of prepregnancy body mass index on pregnancy outcomes, incidence of urinary incontinence and quality of life during pregnancy - an observational cohort study. Biomed J.

[16] Moher D, Shamseer L, Clarke M, Ghersi D, Liberati A, Petticrew M (2015). Preferred reporting items for systematic review and meta-analysis protocols (PRISMA-P) 2015 statement. Syst Rev.

[17] StataCorp. Stata Statistical Software: Release 16. College Station, TX: StataCorp LLC; 2019.

[18] Higgins JP, Thompson SG, Deeks JJ, Altman DG (2003). Measuring inconsistency in meta-analyses. BMJ.

[19] Egger M, Davey Smith G, Schneider M, Minder C (1997). Bias in meta-analysis detected by a simple, graphical test. BMJ.

[20] Sayed Shama EE, Ibrahiem NM, Ahmed AR, El-Berdan A, El-Sherbeny E (2020). Clinical association between gestational diabetes mellitus and quality of life among women. Malays J Nurs.

[21] Halkoaho A, Kavilo M, Pietilä AM, Huopio H, Sintonen H, Heinonen S (2010). Does gestational diabetes affect women’s health-related quality of life after delivery?. Eur J Obstet Gynecol Reprod Biol.

[22] Lee KW, Ching SM, Hoo FK, Ramachandran V, Chong SC, Tusimin M (2020). Factors associated with poor-to-moderate quality of life among pregnant women with gestational diabetes mellitus: a cross-sectional study in Malaysia. Qual Life Res.

[23] Pantzartzis KA, Manolopoulos PP, Paschou SA, Kazakos K, Kotsa K, Goulis DG (2019). Gestational diabetes mellitus and quality of life during the third trimester of pregnancy. Qual Life Res.

[24] Danyliv A, Gillespie P, O’Neill C, Noctor E, O’Dea A, Tierney M (2015). Health related quality of life two to five years after gestational diabetes mellitus: cross-sectional comparative study in the ATLANTIC DIP cohort. BMC Pregnancy Childbirth.

[25] Liu J, Wang S, Leng J, Li J, Huo X, Han L (2020). Impacts of gestational diabetes on quality of life in Chinese pregnant women in urban Tianjin, China. Prim Care Diabetes.

[26] Kutowska J, Gierszewska M, Mieczkowska E, Gebuza G, Kaźmierczak M (2012). Quality of life among women with gestational diabetes mellitus. Med Biol Sci.

[27] Doğan RA, Beji NK (2023). Quality of life and depression conditions of women with gestational diabetes during pregnancy and postpartum period. Rev Bras Ginecol Obstet.

[28] Kopec JA, Ogonowski J, Rahman MM, Miazgowski T (2015). Patient-reported outcomes in women with gestational diabetes: a longitudinal study. Int J Behav Med.

[29] Dalfrà MG, Nicolucci A, Bisson T, Bonsembiante B, Lapolla A (2012). Quality of life in pregnancy and post-partum: a study in diabetic patients. Qual Life Res.

[30] Mautner E, Greimel E, Trutnovsky G, Daghofer F, Egger JW, Lang U (2009). Quality of life outcomes in pregnancy and postpartum complicated by hypertensive disorders, gestational diabetes, and preterm birth. J Psychosom Obstet Gynaecol.

[31] Iwanowicz-Palus G, Zarajczyk M, Pięta B, Bień A (2019). Quality of life, social support, acceptance of illness, and self-efficacy among pregnant women with hyperglycemia. Int J Environ Res Public Health.

[32] Baneh TA, Khomami HM, Mirhadian L, Atrkarroushan Z (2019). The relationship between acceptance of illness and quality of life in mothers with gestational diabetes mellitus. J Pharm Res Int.

[33] Mokhlesi S, Simbar M, Ramezani Tehrani F, Kariman N, Alavi Majd H (2019). Quality of life questionnaire for women with gestational diabetes mellitus (GDMQ-36): development and psychometric properties. BMC Pregnancy Childbirth.

[34] Rumbold AR, Crowther CA (2002). Women’s experiences of being screened for gestational diabetes mellitus. Aust N Z J Obstet Gynaecol.

[35] Lee J, Smith JP. Health, Economic Status, and Aging in High-Income Countries. National Academies Press; 2018.

[36] Sudharsanan N, Bloom DE, Sudharsanan N. The demography of aging in low-and middle-income countries: chronological versus functional perspectives. In: Future Directions for the Demography of Aging: Proceedings of a Workshop. Washington, DC: National Academies of Sciences, Engineering, and Medicine; 2018.

[37] Calman KC (1984). Quality of life in cancer patients--an hypothesis. J Med Ethics.

[38] Karimi M, Brazier J (2016). Health, health-related quality of life, and quality of life: what is the difference?. Pharmacoeconomics.

[39] King CR, Hinds PS. Quality of Life: From Nursing and Patient Perspectives. Jones & Bartlett Publishers; 2011.

[40] Azami-Aghdash S, Gharaee H, Aghaei MH, Derakhshani N (2019). Cardiovascular disease patient’s quality of life in Tabriz city in Iran in 2018. J Community Health Res.

[41] Lin XJ, Lin IM, Fan SY (2013). Methodological issues in measuring health-related quality of life. Tzu Chi Med J.

[42] Sahrakorpi N, Rönö K, Koivusalo SB, Stach-Lempinen B, Eriksson JG, Roine RP (2019). Effect of lifestyle counselling on health-related quality of life in women at high risk for gestational diabetes. Eur J Public Health.

[43] Long Q, Guo J, Zhong Q, Jiang S, Wiley J, Chen JL (2021). General self-efficacy and social support as mediators of the association between perceived stress and quality of life among rural women with previous gestational diabetes mellitus. J Clin Nurs.

[44] Sajid MS, Tonsi A, Baig MK (2008). Health-related quality of life measurement. Int J Health Care Qual Assur.

[45] Marchetti D, Carrozzino D, Fraticelli F, Fulcheri M, Vitacolonna E (2017). Quality of life in women with gestational diabetes mellitus: a systematic review. J Diabetes Res.

[46] Mokhlesi S, Simbar M, Ramezani Tehrani F, Kariman N, Alavi Majd H (2019). Quality of life and gestational diabetes mellitus: a review study. interventions. J Womens Health Reprod Sci.

