# Supplementary material for: Examining the quality of life among pregnant women diagnosed with gestational diabetes mellitus: A systematic review and meta-analysis for women’s health promotion
Source: Health Promot Perspect. 2024 Jul 29;14(2):109–20. doi: 10.34172/hpp.2024.05 (PMC11403342; doi:10.34172/hpp.2024.05)
Supplement: Supplementary file 1 — Search Strategy [file hpp-14-109-s001.pdf]

Mobasseri et al, **Health Promotion Perspectives,** 2024, 14(2), 109-S1
doi: 10.34172/hpp.2024.05
https://hpp.tbzmed.ac.ir

Supplementary 1: Search strategy

PubMed Search Strategy

Set	Strategy	Results
**#1**	(((("Quality of life"[Title/Abstract]) OR ("Health-related quality of life"[Title/Abstract])) OR ("QoL"[Title/Abstract])) OR ("HRQoL"[Title/Abstract])))	387,757
**#2**	('Gestational Diabetes Mellitus'[Title/Abstract]) OR (GDM[Title/Abstract])	15,616
**#3**	**#1 AND #2**	**124**

Scopus Search Strategy

Set #		Results
**1**	(TITLE-ABS-KEY ("Quality of life") OR TITLE-ABS-KEY ("Health-related quality of life") OR TITLE-ABS-KEY ("QoL") OR TITLE-ABS-KEY ("HRQoL"))	720,842
**2**	(TITLE-ABS-KEY ("Gestational Diabetes Mellitus") OR TITLE-ABS-KEY ("GDM"))	20,968
**3**	#1 AND #2	**218**

Search Strategy Web of Science

Set #		Results
**1**	"Quality of life" (Topic) or "Health-related quality of life" (Topic) or "QoL" (Topic) or "HRQoL" (Topic)	530,741
**2**	"Gestational Diabetes Mellitus" (Topic) OR “GDM” (Topic)	19,576
**3**	#1 AND #2	**174**
