# Supplementary material for: Examining the quality of life among pregnant women diagnosed with gestational diabetes mellitus: A systematic review and meta-analysis for women’s health promotion
Source: Health Promot Perspect. 2024 Jul 29;14(2):109–20. doi: 10.34172/hpp.2024.05 (PMC11403342; doi:10.34172/hpp.2024.05)

Mobasseri et al, **Health Promotion Perspectives,** 2024, 14(2), 109-S2
doi: 10.34172/hpp.2024.05
https://hpp.tbzmed.ac.ir

Supplementary file 2: Funnel charts to measure the possibility of publication bias in the results of articles

A- Funnel plot for WHOQOL-BREF

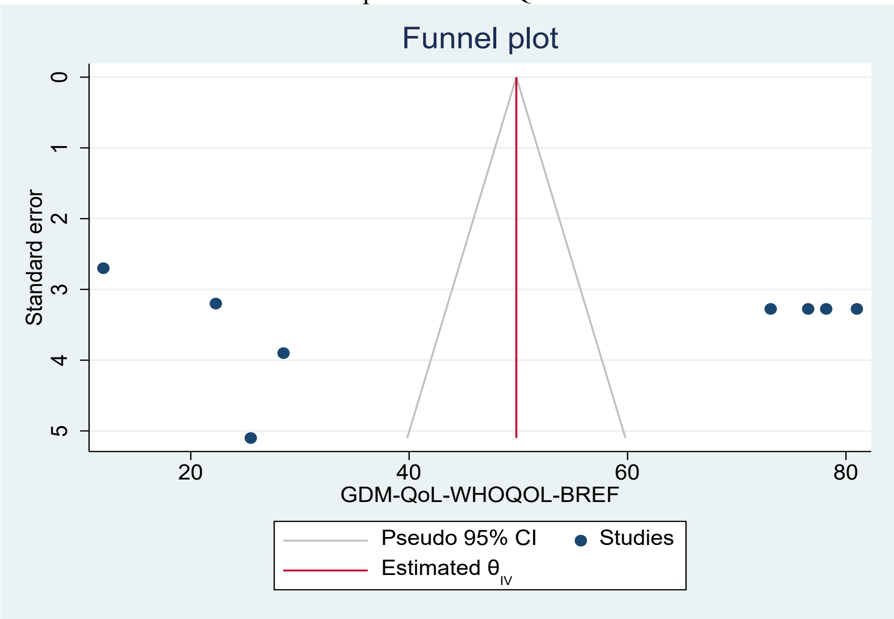

B- Funnel plot for EQ-5D-5L

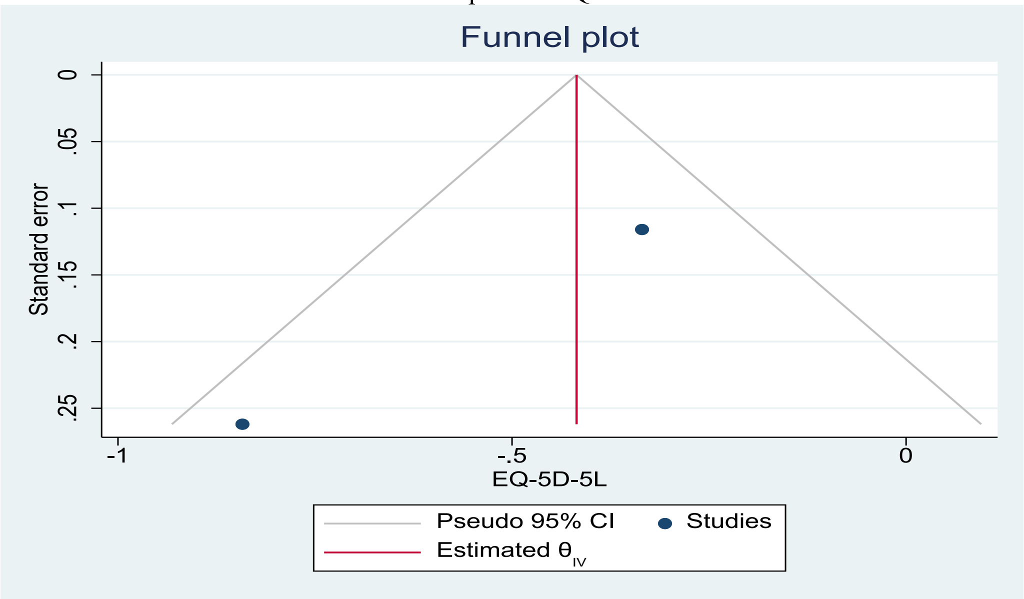

Supplement: Supplementary file 2 — Funnel charts to measure the possibility of publication bias in the results of articles [file hpp-14-109-s002.pdf]
