# Supplementary material for: Examining the quality of life among pregnant women diagnosed with gestational diabetes mellitus: A systematic review and meta-analysis for women’s health promotion
Source: Health Promot Perspect. 2024 Jul 29;14(2):109–20. doi: 10.34172/hpp.2024.05 (PMC11403342; doi:10.34172/hpp.2024.05)
Supplement: Supplementary file 3 — The results of the evaluation of the quality of reporting articles [file hpp-14-109-s003.pdf]

Mobasseri et al, **Health Promotion Perspectives**, 2024, 14(2), 109-S3
doi: 10.34172/hpp.2024.05
https://hpp.tbzmed.ac.ir

Supplementary file 3: The results of the evaluation of the quality of reporting articles

reference	1	2	3	4	5	6	7	Total score
1. (1)	YES:<br>NO: ■<br>Unclear:<br>NA:	YES: ■<br>NO:<br>Unclear:<br>NA:	YES:<br>NO:<br>Unclear: ■<br>NA:	YES:<br>NO: ■<br>Unclear:<br>NA:	YES:<br>NO: ■<br>Unclear:<br>NA:	YES: ■<br>NO:<br>Unclear:<br>NA:	YES: ■<br>NO:<br>Unclear:<br>NA:	3
2. (2)	YES: ■<br>NO:<br>Unclear:<br>NA:	YES: ■<br>NO:<br>Unclear:<br>NA:	YES:<br>NO:<br>Unclear: ■<br>NA:	YES:<br>NO:<br>Unclear: ■<br>NA:	YES:<br>NO:<br>Unclear: ■<br>NA:	YES: ■<br>NO:<br>Unclear:<br>NA:	YES: ■<br>NO:<br>Unclear:<br>NA:	4
3. (3)	YES: ■<br>NO:<br>Unclear:<br>NA:	YES: ■<br>NO:<br>Unclear:<br>NA:	YES: ■<br>NO:<br>Unclear:<br>NA:	YES:<br>NO:<br>Unclear: ■<br>NA:	YES:<br>NO:<br>Unclear: ■<br>NA:	YES: ■<br>NO:<br>Unclear:<br>NA:	YES: ■<br>NO:<br>Unclear:<br>NA:	5
4. (4)	YES: ■<br>NO:<br>Unclear:<br>NA:	YES: ■<br>NO:<br>Unclear:<br>NA:	YES: ■<br>NO:<br>Unclear:<br>NA:	YES:<br>NO:<br>Unclear: ■<br>NA:	YES:<br>NO:<br>Unclear: ■<br>NA:	YES: ■<br>NO:<br>Unclear:<br>NA:	YES: ■<br>NO:<br>Unclear:<br>NA:	5
5. (5)	YES: ■<br>NO:<br>Unclear:<br>NA:	YES: ■<br>NO:<br>Unclear:<br>NA:	YES: ■<br>NO:<br>Unclear:<br>NA:	YES: ■<br>NO:<br>Unclear:<br>NA:	YES: ■<br>NO:<br>Unclear:<br>NA:	YES: ■<br>NO:<br>Unclear:<br>NA:	YES: ■<br>NO:<br>Unclear:<br>NA:	7
6. (6)	YES: ■<br>NO:<br>Unclear:<br>NA:	YES: ■<br>NO:<br>Unclear:<br>NA:	YES: ■<br>NO:<br>Unclear:<br>NA:	YES: ■<br>NO:<br>Unclear:<br>NA:	YES: ■<br>NO:<br>Unclear:<br>NA:	YES: ■<br>NO:<br>Unclear:<br>NA:	YES: ■<br>NO:<br>Unclear:<br>NA:	7
7. (7)	YES:<br>NO: ■<br>Unclear:<br>NA:	YES: ■<br>NO:<br>Unclear:<br>NA:	YES:<br>NO:<br>Unclear: ■<br>NA:	YES:<br>NO: ■<br>Unclear:<br>NA:	YES:<br>NO: ■<br>Unclear:<br>NA:	YES: ■<br>NO:<br>Unclear:<br>NA:	YES:<br>NO: ■<br>Unclear:<br>NA:	2
8. (8)	YES: ■<br>NO:<br>Unclear:<br>NA:	YES: ■<br>NO:<br>Unclear:<br>NA:	YES: ■<br>NO:<br>Unclear:<br>NA:	YES:<br>NO:<br>Unclear: ■<br>NA:	YES:<br>NO:<br>Unclear: ■<br>NA:	YES: ■<br>NO:<br>Unclear:<br>NA:	YES: ■<br>NO:<br>Unclear:<br>NA:	5
9. (9)	YES:<br>NO:<br>Unclear: ■<br>NA:	YES: ■<br>NO:<br>Unclear:<br>NA:	YES: ■<br>NO:<br>Unclear:<br>NA:	YES:<br>NO:<br>Unclear: ■<br>NA:	YES:<br>NO:<br>Unclear: ■<br>NA:	YES: ■<br>NO:<br>Unclear:<br>NA:	YES: ■<br>NO:<br>Unclear:<br>NA:	4
10. (10)	YES: ■<br>NO:<br>Unclear:<br>NA:	YES: ■<br>NO:<br>Unclear:<br>NA:	YES: ■<br>NO:<br>Unclear:<br>NA:	YES:<br>NO:<br>Unclear: ■<br>NA:	YES:<br>NO:<br>Unclear: ■<br>NA:	YES: ■<br>NO:<br>Unclear:<br>NA:	YES: ■<br>NO:<br>Unclear:<br>NA:	5

11. (11)	YES: ■<br>NO:<br>Unclear:<br>NA:	YES: ■<br>NO:<br>Unclear:<br>NA:	YES: ■<br>NO:<br>Unclear:<br>NA:	YES:<br>NO:<br>Unclear: ■<br>NA:	YES:<br>NO:<br>Unclear: ■<br>NA:	YES: ■<br>NO:<br>Unclear:<br>NA:	YES: ■<br>NO:<br>Unclear:<br>NA:	5
12. (12)	YES: ■<br>NO:<br>Unclear:<br>NA:	YES: ■<br>NO:<br>Unclear:<br>NA:	YES: ■<br>NO:<br>Unclear:<br>NA:	YES: ■<br>NO:<br>Unclear:<br>NA:	YES:<br>NO:<br>Unclear: ■<br>NA:	YES: ■<br>NO:<br>Unclear:<br>NA:	YES: ■<br>NO:<br>Unclear:<br>NA:	6
13. (13)	YES: ■<br>NO:<br>Unclear:<br>NA:	YES: ■<br>NO:<br>Unclear:<br>NA:	YES: ■<br>NO:<br>Unclear:<br>NA:	YES:<br>NO:<br>Unclear: ■<br>NA:	YES:<br>NO:<br>Unclear: ■<br>NA:	YES: ■<br>NO:<br>Unclear:<br>NA:	YES: ■<br>NO:<br>Unclear:<br>NA:	5
14. (14)	YES: ■<br>NO:<br>Unclear:<br>NA:	YES: ■<br>NO:<br>Unclear:<br>NA:	YES: ■<br>NO:<br>Unclear:<br>NA:	YES:<br>NO:<br>Unclear: ■<br>NA:	YES:<br>NO:<br>Unclear: ■<br>NA:	YES:<br>NO:<br>Unclear: ■<br>NA:	YES: ■<br>NO:<br>Unclear:<br>NA:	4
15. (15)	YES: ■<br>NO:<br>Unclear:<br>NA:	YES: ■<br>NO:<br>Unclear:<br>NA:	YES: ■<br>NO:<br>Unclear:<br>NA:	YES:<br>NO:<br>Unclear: ■<br>NA:	YES:<br>NO:<br>Unclear: ■<br>NA:	YES: ■<br>NO:<br>Unclear:<br>NA:	YES: ■<br>NO:<br>Unclear:<br>NA:	5

1. Were the criteria for inclusion in the sample clearly defined? 2. Were the study subjects and the setting described in detail? 3. Was the exposure measured in a valid and reliable way? 4. Were objective, standard criteria used for measurement of the condition? 5. Were confounding factors identified? 6. Were strategies to deal with confounding factors stated? 7. Were the outcomes measured in a valid and reliable way? 8. Was appropriate statistical analysis used?
